# Impact of satisfaction with physician–patient communication on self-care and adherence in patients with hypertension: cross-sectional study

**DOI:** 10.1186/s12913-020-05912-0

**Published:** 2020-11-16

**Authors:** Natalia Świątoniowska-Lonc, Jacek Polański, Wojciech Tański, Beata Jankowska-Polańska

**Affiliations:** 1 Department of Clinical Nursing, Faculty of Health Science, Wroclaw Medical University, K. Bartla 5, 51-616, Wroclaw, Poland; 2 Faculty of Medicine, Wroclaw Medical University, Wroclaw, Poland; 3 Department of Internal Medicine, 4th Military Teaching Hospital, Wroclaw, Poland

**Keywords:** Adherence, Communication, Self-care, Hypertension

## Abstract

**Background:**

Hypertension (HT) requires patients to continuously monitor their blood pressure, strictly adhere to therapeutic recommendations, and self-manage their illness. A few studies indicate that physician–patient communication and the patient’s satisfaction with the therapeutic relationship may affect the course and outcomes of the treatment process. Research is still lacking on the association between satisfaction with physician–patient communication and adherence to treatment or self-care in chronically ill patients. The aim of the study was to evaluate the relationship between satisfaction with physician–patient communication and self-care and adherence in patients with HT undergoing chronic treatment.

**Methods:**

The following instruments were used: the Adherence to Refills and Medication Scale (ARMS) for evaluating adherence (12–48 points), the Self-Care of Hypertension Inventory (SCHI) for self-care level (0–100 points), and the Communication Assessment Tool (CAT) for evaluating satisfaction with physician–patient communication. Socio-demographic and clinical data were obtained from patients’ medical records. The research has a cross-sectional and observational study design. Inclusion criteria were as follows: age > 18 years, hypertension diagnosed per European Society of Hypertension (ESH) guidelines, treatment with at least one antihypertensive drug for the past 6 months, and informed consent. Cognitively impaired patients unable to complete the surveys without assistance were excluded (MMSE ≤18). Correlations between quantitative variables were analyzed using Pearson’s or Spearman’s correlation coefficient. Linear regression was performed. Variable distribution normality was verified using the Shapiro-Wilk test.

**Results:**

The study included 250 patients (110 male, mean age 61.23 ± 14.34) with HT, treated at a hypertension clinic. In the CAT questionnaire individual questions pertaining to satisfaction with physician communication (on the CAT) were rated “excellent” 28.4–50.4% of the time. The best-rated aspects of communication included: letting the patient talk without interruptions (50.4% “excellent” ratings), speaking in a way the patient can understand (47.6%), and paying attention to the patient (47.2%). According to patient reports, physicians most commonly omitted such aspects as encouraging the patient to ask questions (28.4%), involving them in decisions (29.2%), and discussing the next steps (35.2%). The respondents had a low level of adherence to pharmaceutical treatment (16.63 ± 4.6). In terms of self-care, they scored highest in self-care management (64.17 ± 21.18), and lowest in self-care maintenance (56.73 ± 18.57). In correlation analysis, satisfaction with physician–patient communication (total CAT score) was positively correlated with all SCHI domains (self-care maintenance β = 0.276, self-care management β = 0.208, self-care confidence β = 0.286, *p* < 0.05), and negatively correlated with ARMS scores (indicating better adherence).

**Conclusions:**

Satisfaction with physician–patient communication has a significant impact on self-care and pharmaceutical adherence in patients with hypertension. The more satisfied the patient is with communication, the better their adherence and self-care.

**Trial registration:**

SIMPLE: RID.Z501.19.016.

## Background

Hypertension (HT) is a global health and economic problem [1]. According to WHO data, high blood pressure (BP) accounts for 13% of all deaths worldwide [1]. By 2025, an estimated 1.5 billion people worldwide will have HT [2]. Untreated HT leads to a number of debilitating complications such as chronic heart disease, stroke, coronary heart disease, retinopathy and reduced kidney function [3]. HT treatment is most effective when the patient is cooperative and fully involved in the treatment process, which includes adhering to treatment, performing self-care, and monitoring signs of high blood pressure [4].

Self-care is a key element in the long-term management of chronic diseases and is defined as a process of maintaining health through health-promoting practices and disease management [5, 6]. Selfcare can be seen as an overarching structure built on 3 key concepts of self-care maintenance (e.g. observing self-care behaviors such as regular exercise and taking medication as recommended), monitoring (e.g. regular change measurements, routine tests) and management (e.g. change of diet or medication dose based on detection and interpretation of 71 symptoms). The 3 concepts of self-care maintenance, monitoring, and management are closely related; therefore, sufficient self-care includes all three behaviors [5, 6]. Though evidence-based self-care behaviors in HT allow for normalizing BP, patients with HT do not typically adhere fully to these behaviors. In case of primary hypertension self-care involves lifestyle changes, including proper diet and weight control, and pharmaceutical adherence [7, 8]. Together, these behaviors contribute to better BP control and symptom monitoring. Poor patient cooperation in the treatment process is associated with poor outcomes such as patient recall, patient understanding, and patient adherence to therapy [9]. Non-adherence to treatment is the most common cause of treatment failure. Research has identified multiple factors with an impact on adherence and self-care [6, 10–14]. The WHO has defined five groups of factors contributing to non-adherence: (1) patient and family-dependent factors, (2) illness-related factors, (3) treatment-related factors, (4) healthcare system-related factors, and (5) socio-demographic and economic factors [15]. Healthcare system-related factors include communication and satisfaction with treatment.

The role of socio-demographic and clinical, patient-related, and illness-related factors is studied quite often [16, 17]. Research is still lacking, however, on factors related to the healthcare system, and in particular, satisfaction with treatment and with physician–patient communication. Physician-patient communication has a number of functions, including exchanging information, managing the patient’s uncertainty, promoting self-management, addressing emotions, improving the physician–patient relationship, and making decisions [18]. For patients, what matters in the communication process is a sense of being a partner in their treatment process, and a feeling of having their needs understood by medical personnel [19].

Effective physician-provider communication results in better psychological, somatic and social health [20]. In the communication process, the physician may promote positive motivations and patient involvement in the treatment. The patient must understand their illness, the associated risks, and the benefits of consistent treatment. It is important to understand the preferences and beliefs and perspectives of patient health evaluation. In the therapeutic process, the doctor and the patient do not always gain mutual understanding, and their expectations, perspectives may be completely different. The process of mutual communication aims to stimulate or strengthen the sense of control over health, the ability to recognize symptoms and self-care. It is important to note that communication should produce or strengthen a sense of control over the illness, and an ability to identify symptoms and changes in one’s condition [21].

The available data [14, 22–25], which come mainly from the US, document the role of communication in the physician–patient relationship. Few European reports are available [14, 25], and to the best of our knowledge, there have been no Polish studies focusing on the relationship between communication and adherence or self-care in patients with HT.

## Methods

### Aim

The aim of the study was to evaluate the relationship between satisfaction with physician–patient communication and self-care and adherence in patients with HT undergoing chronic treatment.

The primary outcome of our study was the perceived quality of the physician–patient communication. The secondary outcome was the relationship between physician–patient communication on the one hand, and pharmaceutical adherence and self-care on the other.

### Design

The present research has a cross-sectional and observational study design. Data were collected between January 2019 and August 2019 from patients who reported for follow-up appointments at the clinical division of internal medicine with specialization in hypertension. The study used a closed-ended standardized survey.

## Data collection

### Participants

The study included 250 patients (110 male) diagnosed with HT per European Society of Cardiology (ESC) guidelines [4].

Inclusion criteria were as follows: age above 18 years, hypertension diagnosed per European Society of Hypertension (ESH) guidelines, treatment with at least one antihypertensive drug for the past 6 months, and informed consent. Patients with exacerbations of other serious diseases, which could affect adherence to treatment and/or interfere with survey completion, were excluded from the study. Cognitively impaired patients unable to complete the surveys without assistance were also excluded (patients aged 65 or more were tested using the MMSE questionnaire, and those with scores ≤18 were excluded from the study).

Patients were selected by a panel consisting of a physician and a nurse-specialist in the field of internal medicine. The personnel were informed about the aim of the study. A study protocol was prepared for the purpose of the study so that the personnel could collect data in the same way. Respondents answered all questions directly, based on their last 4 weeks of treatment. Patients filled in the questionnaire on their own in the paper version. Patients not able to complete the questionnaire on their own due to their health condition were excluded. The nurse was always available and helpful but did not fill in the questionnaires with the patient. Patients were informed that they could contact a nurse specialist (available in the clinic) if they needed any clarification or guidance related to the survey. Socio-demographic and clinical data were obtained from the patients’ medical records, with their consent.

In the study period (January–August 2019), 323 patients with hypertension diagnosed in accordance with the ESC criteria were hospitalized in the department. In this group, 40 patients did not meet the inclusion criteria, and 26 refused to participate. Therefore, 257 patients were included in the study and received surveys; however, during the study, 7 patients dropped out without providing a reason or did not complete the survey correctly. The final group included 250 patients. All patients were informed about the study course and methods, and about the possibility of withdrawing from the study at any time.

### Instruments

The following standardized questionnaires were used:

(1) The Communication Assessment Tool (CAT), comprising 14 items for evaluating the quality of specific aspects of physician–patient communication. Responses are provided using a 5-item Likert scale, from 1 (poor) to 5 (excellent). Higher percentages of “excellent” scores indicate more satisfaction with communication. The original version of the Communication Assessment Tool is internally consistent, with a high scale reliability (Cronbach’s coefficient alpha = 0.98) [26].

(2) The Adherence to Refills and Medication Scale (ARMS), which evaluates the patient’s adherence level. It comprises 12 items related to various aspects of non-adherence, scored on the following scale: 1 — never, 2 — rarely, 3 — often, 4 — most of the time. Therefore, total scores range between 12 and 48 points, with higher scores indicating poorer adherence. In the polish version of ARMS-12 reliability analysis, standardized Cronbach’s α was 0.954 [11].

(3) The Self-Care of Hypertension Inventory (SCHI), which enables the evaluation of a hypertensive patient’s independence in three aspects of daily functioning with the illness: self-management, self-maintenance, and self-confidence. Scores in each aspect range from 0 to 100, with higher scores indicating more independence in the relevant aspect. In the original version of SCHI a unidimensional confidence factor captured confidence in and persistence with each aspect of self-care (α = 0.83) [5].

### Ethical consideration

The study was approved by the local Bioethics Committee (approval no. KB 42/2019). All participants provided written informed consent after a thorough explanation of all procedures involved. All patients received information about the purpose and nature of the study and provided written informed consent to participate. All patients completed all questionnaires. The study was carried out in accordance with the tenets of the Declaration of Helsinki.

### Statistical analysis

Patients were broken down into two groups: group 1 — poor communication (*n* = 34), group 2 — good communication (*n* = 216).

Correlations between quantitative variables were analyzed using Pearson’s correlation coefficient (if distributions for both were normal) or Spearman’s correlation coefficient (otherwise) [27]. Multivariate analysis of the independent impact of the selected variables on the quantitative variable was performed using linear regression. The results are shown as regression model parameter values with a 95% confidence interval (CI). Variable distribution normality was verified using the Shapiro-Wilk test. All analyses used a significance threshold of 0.05. i.e. all *p* values of less than 0.05 were interpreted as showing significant associations. The analyses were performed using the R software, version 3.6.0 [28].

## Results

### Socio-demographic and clinical characteristics of the group

The study included 250 patients (110 male) with a mean age of 61.23 ± 14.34 years. Comparative analysis demonstrated significant differences in patients’ socio-demographic and clinical characteristics (Table 1). Patients were divided into two groups according to the sum of answers given - poor communication (0–42 points), good communication (43–70 points). Patients reporting poor communication with the physician were older (65.68 ± 10.69 vs. 60.53 ± 14.73 years), spent less time in an appointment (16.21 ± 9.52 vs. 20.08 ± 10.13 min), and discussed their problems for a shorter time during an appointment (7.38 ± 7.22 vs. 10.43 ± 9.39 min) compared to those reporting good physician–patient communication. The groups did not differ in terms of the remaining variables.

**Table 1:** Socio-demographic and clinical characteristics of the study group

Variable		Poor communication (***n*** = 34)	Good communication (***n*** = 216)	***p***
Age	mean ± SD	65.68 ± 10.69	60.53 ± 14.73	0.049 NP
Duration of HT in years	mean ± SD	10.24 ± 10.77	12.4 ± 10.34	0.124 NP
Appointment duration [min]	mean ± SD	16.21 ± 9.52	20.08 ± 10.13	0.008 NP
Time spent discussing the patient’s problems [min]	mean ± SD	7.38 ± 7.22	10.43 ± 9.39	0.037 NP
Sex	Female	18 (52.94%)	122 (56.48%)	0.841
	Male	16 (47.06%)	94 (43.52%)	chi2
Place of residence	Rural	6 (17.65%)	51 (23.61%)	0.582
	Urban	28 (82.35%)	165 (76.39%)	chi2
Relationship status	Single	9 (26.47%)	65 (30.09%)	0.82
	In a relationship	25 (73.53%)	151 (69.91%)	chi2
Education	Primary or none	3 (8.82%)	20 (9.26%)	0.762
	High school	20 (58.82%)	112 (51.85%)	F
	College/university	11 (32.35%)	84 (38.89%)	
Professional status	Professionally active	9 (26.47%)	77 (35.65%)	0.338
	Retirement pensioner	20 (58.82%)	96 (44.44%)	F
	Disability pensioner	3 (8.82%)	34 (15.74%)	
	Unemployed	2 (5.88%)	9 (4.17%)	
Financial standing	Wealthy	0 (0.00%)	12 (5.56%)	0.723
	Able to afford all that is needed and save some money	14 (41.18%)	83 (38.43%)	F
	Able to afford daily expenses, but not any larger ones	17 (50.00%)	93 (43.06%)	
	Unable to afford many things	3 (8.82%)	25 (11.57%)	
	Unable to afford even the most basic expenses	0 (0.00%)	3 (1.39%)	
Frequency of follow-up appointments	1	3 (8.82%)	12 (5.56%)	0.557
	1–2	4 (11.76%)	39 (18.06%)	F
	4–5	9 (26.47%)	38 (17.59%)	
	More than 5	18 (52.94%)	124 (57.41%)	
	None	0 (0.00%)	3 (1.39%)	
BMI:	Normal weight	7 (20.59%)	65 (30.09%)	0.514
	Overweight	15 (44.12%)	81 (37.50%)	chi2
	Obesity	12 (35.29%)	70 (32.41%)	
BP	Normal BP	22 (64.71%)	120 (55.56%)	0.415
	Elevated BP	12 (35.29%)	96 (44.44%)	chi2

*P* normal (parametric) distribution in groups, Student’s t-test; *NP* non-parametric distribution in groups, Mann-Whitney test, *BP* blood pressure, *HT* hypertension, *BMI* Body Mass Index

### Satisfaction with physician–patient communication

In the CAT questionnaire individual questions pertaining to satisfaction with physician communication (on the CAT) were rated “excellent” 28.4–50.4% of the time. The best-rated aspects of communication included: letting the patient talk without interruptions (50.4% “excellent” ratings), speaking in a way the patient can understand (47.6%) and paying attention to the patient (47.2%). According to patient reports, physicians most commonly omitted such aspects as encouraging the patient to ask questions (28.4%), involving them in decisions (29.2%), and discussing next steps (35.2% — Table 2).

**Table 2:** CAT scores

Item		% of “excellent” scores
1	The physician greeted me in a way that made me feel comfortable	38.4%
2	The physician treated me with respect	46.0%
3	The physician showed interest in my ideas about my health	44.8%
4	The physician understood my main health concerns	44.8%
5	The physician paid attention to me (looked at me, listened carefully)	47.2%
6	The physician let me talk without interruptions	50.4%
7	The physician gave me all the information I wanted	40.8%
8	The physician talked in terms I could understand	47.6%
9	The physician checked to be sure I understood everything	39.2%
10	The physician encouraged me to ask questions	28.4%
11	The physician involved me in decisions	29.2%
12	The physician discussed next steps, including any follow-up plans	35.2%
13	The physician showed care and concern	42.0%
14	The physician spent the right amount of time with me	42.3%

### Self-care and adherence to pharmaceutical treatment depending on satisfaction with communication

SCHI scores in the entire group showed that patients fared best in terms of self-care management (64.17 ± 21.18), and worst in terms of self-care maintenance (56.73 ± 18.57) (Table 3). The evaluation of pharmaceutical adherence levels using the ARMS questionnaire demonstrated good adherence in the group studied (Table 3). The mean score on the ARMS questionnaire was 16.63 (SD = 4.6). The mean score per item was 1.39, indicating that most patients “never” or “rarely” failed to adhere to treatment.

**Table 3:** Comparison of self-care and adherence levels between groups broken down by satisfaction with patient-provider communication

SC-HI	All patients ***N*** = 250		Poor communication (CAT 0–42)***N*** = 34		Good communication (CAT 43–70) ***N*** = 216		***p***
	Mean	SD	Mean	SD	Mean	SD	
Self-care maintenance	56.73	18.57	53.12	16.67	57.3	18.82	0.224*
Self-care management	64.17	21.18	56.62	22.54	65.36	20.77	0.059**
Self-care confidence	62.47	24.39	51.47	26.03	64.2	23.72	0.005**
**ARMS [points]**	16.63	4.6	18.88	5.76	16.28	4.3	0.104**

* Normal distribution in groups, Student’s t-test; ** lack of normal distribution in groups, *Mann* Whitney test, *SD* standard deviation, *SC-HI* The Self-Care of Hypertension Inventory, *ARMS* The Adherence to Refills and Medication Scale, *CAT* The Communication Assessment

When comparing results between groups broken down by patient-provider communication quality, higher self-care and adherence levels were found in the “good communication” group, with statistically significant differences between the two groups in terms of self-care confidence (*p* = 0.005), and not significant differences in terms of self-care management (*p* = 0.059).

Self-care in the specific domains was correlated with odds of good communication. Factors with an impact on this parameter included self-care management (OR = 1.019), self-care confidence (OR = 1.021), and adherence score in the ARMS (OR = 0.905) (Table 4). Each additional point in the self-care management domain increased the odds of good communication by 1.9%, and in the self-care confidence domain — by 2.1%. Higher scores in the ARMS (i.e. poorer adherence) decreased the odds of good communication by 9.5%.

**Table 4:** Odds ratios for good communication

Variable	OR	95% CI		***p*** *
SC-HI: Self-care maintenance	1.012	0.993	1.033	0.224
SC-HI: Self-care management	1.019	1.002	1.036	0.027
SC-HI: Self-care confidence	1.021	1.006	1.036	0.006
ARMS	0.905	0.847	0.968	0.004

* single-factor logistic regression, *OR* odds ratio, *CI* confidence interval, *SC-HI* The Self-Care of Hypertension Inventory, *ARMS* The Adherence to Refills and Medication Scale, *CAT* The Communication Assessment

### Impact of communication on self-care and adherence

In the correlation analysis, satisfaction with communication scores on the CAT questionnaire were significantly positively correlated with all SCHI domains (self-care maintenance *r* = 0.208, self-care management *r* = 0.276, self-care confidence *r* = 0.284), i.e. more satisfaction with communication was associated with better self-care in all SCHI domains (Table 4). The correlation between satisfaction with communication and pharmaceutical adherence (ARMS score) is statistically significant and negative (*r* = − 0.299, *p* < 0.001), i.e. more satisfaction with communication was associated with lower ARMS score, indicating better adherence (Table 5).

**Table 5:** Correlation analysis for CAT, SCHI, and ARMS scores

Parameter		Correlation with CAT			
		Correlation coefficient	***p*** *	Correlation direction	Correlation strength
SC-HI	Self-care maintenance	0.208	*p* = 0.001 NP	positive	very weak
	Self-care management	0.276	*p* < 0.001 NP	positive	very weak
	Self-care confidence	0.284	*p* < 0.001 NP	positive	very weak
	ARMS [points]	−0.299	*p* < 0.001 NP	negative	very weak

* *P* = normal (parametric) distribution of both correlated variables, Pearson’s correlation coefficient used, *NP* = non-parametric distribution for at least one of the correlated variables, Spearman’s correlation coefficient used, *CAT* The Communication Assessment, *SC-HI* The Self-Care of Hypertension Inventory, *ARMS* The Adherence to Refills and Medication Scale

## Discussion

To the best of our knowledge, ours is the first study (based on polish patients) to identify communication between the physician and the patient as a significant predictor of adherence to pharmaceutical treatment and of self-care in patients with HT.

In our study, patients had the best results in self-management, and the most difficulties in self-maintenance. Research shows an association between self-maintenance and good BP control [29]. In a study by Logan et al., 51% of patients in a group provided with education on BP measurement achieved the target of < 130/80 mmHg, compared to 31% in the control group [30]. According to Cheng, patients are ambivalent about measuring their BP at home: despite access to a BP meter and encouragement from their physician, only a third actually report their home readings to the physician. What is worse, as many as 40% take no action whatsoever to normalize their BP [31]. In other studies, the percentage of hypertensive patients with good self-care practices ranged between 20.3 and 37.1% [8, 32]. In their study of patients with HT, Gebremichael et al. demonstrated an association between good self-care practices and good BP control [32].

Our study was performed in a secondary care setting, where patients are seen less frequently. A large proportion of care is provided in primary care settings. This is the first line of monitoring the patients’ health, with broader possibilities of providing constant care, monitoring lifestyle changes, and correcting patients’ behaviors. In our study, discussing the next steps or follow-up plans was among the most commonly omitted aspects of the appointments (35.2%). Mendes et al. compared self-care levels of HT patients in primary and secondary healthcare settings. Patients using primary care services adhered to fluid restrictions and kept follow-up appointments significantly more often than those in secondary care. Other self-care components did not differ between the groups [10]. One could infer that patients treated in a secondary care setting have a more advanced clinical condition (complications, multimorbidity), warranting the higher referral level.

In our study, the mean pharmaceutical adherence score was 16.63 ± 4.6, indicating satisfactory adherence. Compared to other Polish studies on HT patients, we found higher adherence levels based on the patient profile — female sex, age below 65, being in a relationship, having a high school or college/university education, and normal BP levels [11–13, 16]. In the literature, there are many studies on adherence among patients with HT. Nearly 50% of patients do not take their medications as prescribed [1]. Non-adherence to HT treatment results in uncontrolled BP [11, 12]. Compared to literature reports, we found more satisfactory adherence levels — in the literature, more than half of patients do not take their medication, and even among those who do, the results are poor [16].

Communication between health care personnel and the patient has a significant impact on the patient’s attitude towards their illness [33]. The primary aim of our study was to evaluate patients’ satisfaction with communication and its impact on their involvement in the treatment process. Studies on patients with diabetes mellitus confirm the impact of communication on better adherence and self-care in chronically-ill patients, but also that of adherence and self-care on better physician–patient communication [34–36]. In a meta-analysis performed by Zolnierek et al., the non-adherence percentage was 19% higher in those patients who reported poor physician–patient communication [9]. In our study, satisfaction with communication was significantly correlated with all self-care domains and with adherence. In published studies, communication interventions, though differing in methods and content, did affect chronically-ill patients’ adherence to treatment [34, 37]. Heisler et al. demonstrated that a higher level of explanatory physician communication was significantly associated with more independence in medication-taking among elderly patients [34]. In addition to diagnosis and treatment, patients expect their primary care physicians to be competent in contact and communication. Patients appreciate the value of talking to their physician, and some even consider it therapeutic in itself [21]. In our study, patients were satisfied with the clarity of communication, the ability to speak without interruption, and the amount of attention they received. Patients value physicians who are good listeners. A physician-patient communication style requires including the patient in treatment planning [21, 38]. In their relationship with the primary care physician, patients may prefer a “partnership” model of care, and some physicians do demonstrate this kind of attitude. However, not all physicians approve of the patient’s active participation in consultations [21]. The patients in our study complained of not being included in decision-making (29.2%). Physician-patient communication may contribute to joint decisions about the treatment. Patients’ active attitude and physician-patient communication may also provide the physician with information on the perceived advantages and disadvantages of the proposed treatment course [35]. Ratanawongsa et al. demonstrated an association between a lower level of adherence to refills and physician characteristics such as poor ability to involve patients in decision-making, lack of understanding of patients’ problems associated with the treatment, and failure to inspire trust and confidence [36]. In our study, patients most commonly reported not being encouraged to ask questions (only 28.4% of “excellent” answers). For the physician, patients’ answers to their questions are the main source of information. As some patients may lack the courage to ask questions themselves, the physician should encourage them and make them feel comfortable enough to ask questions freely.

Our findings show that the entire communication process, with all its components, plays a role in improving self-care and adherence. Correlation analysis for specific items on the questionnaire did not demonstrate any statistically significant impact on self-care and adherence results, except for the self-management domain. It seems, then, that it is an overall perception of communication as a whole, rather than any specific component, that affects patients’ self-care and adherence.

## Study limitations

Our study had a few limitations. One is the lack of a health literacy assessment. Patients’ knowledge on health and disease may be a factor facilitating active participation in consultations with a physician [39]. Patients with a low level of health literacy have difficulties communicating with medical personnel [40]. In a study by Ishikawa et al., a more critical approach to analyzing information on diabetes was associated with more knowledge on the illness, a larger number of information sources, and more independence in terms of diabetes self-care, while a higher level of health literacy in the communication domain was correlated with better physician–patient communication during consultations [41]. Another limitation is not analyzing the impact of the patients’ sex on physician–patient communication. Women are more likely than men to discuss their problems with their physician, and are more willing to undergo treatment. The sex of the physician could also be a factor. Patients are more willing to talk to female physicians, who in turn are more empathetic towards patients [22].

## Conclusions

Patients with HT treated at a specialist clinic report a high level of satisfaction with communication. The most important aspects from the patients’ perspective include the physician speaking in an understandable way and paying attention. The most common complaints include not being encouraged to ask questions and not being included in the treatment plan and decision-making. Satisfaction with physician–patient communication is significantly correlated with better self-care and pharmaceutical adherence in patients with HT. Patients with HT demonstrate a high level of pharmaceutical adherence and self-care skills, especially in terms of self-care management. The lowest self-care quality was found in terms of self-maintenance.

## Data Availability

The datasets used and/or analyzed during the current study are available from the corresponding author on reasonable request.

## Abbreviations

ARMS: The Adherence to Refills and Medication Scale
BMI: Body Mass Index
BP: Blood pressure
CAT: The Communication Assessment Tool
CI: 95% confidence interval
ESC: The European Society of Cardiology
ESH: The European Society of Hypertension
HT: Hypertension
MMSE: The Mini-Mental State Examination
SCHI: The Self-Care of Hypertension Inventory
SD: Standard deviation
US: The United States
WHO: World Health Organization
